# Regulation of post-translational modification of PD-L1 and advances in tumor immunotherapy

**DOI:** 10.3389/fimmu.2023.1230135

**Published:** 2023-07-24

**Authors:** Chong Feng, Lening Zhang, Xin Chang, Dongliang Qin, Tao Zhang

**Affiliations:** ^1^ Thoracic Surgery, China-Japan Union Hospital of Jilin University, Changchun, Jilin, China; ^2^ Ophthalmology, China-Japan Union Hospital of Jilin University, Changchun, Jilin, China; ^3^ Gastrointestinal and Colorectal Surgery, China-Japan Union Hospital of Jilin University, Changchun, Jilin, China

**Keywords:** post-translational modification, tumor immunotherapy, programmed death ligand 1, glycosylation, ubiquitination, phosphorylation, acetylation, S-palmitoylation

## Abstract

The immune checkpoint molecules programmed cell death receptor 1 (PD-1) and programmed death ligand 1 (PD-L1) are one of the most promising targets for tumor immunotherapy. PD-L1 is overexpressed on the surface of tumor cells and inhibits T cell activation upon binding to PD⁃1 on the surface of T cells, resulting in tumor immune escape. The therapeutic strategy of targeting PD-1/PD-L1 involves blocking this binding and restoring the tumor-killing effect of immune cells. However, in clinical settings, a relatively low proportion of cancer patients have responded well to PD-1/PD-L1 blockade, and clinical outcomes have reached a bottleneck and no substantial progress has been made. In recent years, PD-L1 post-translation modifications (PTMs) have gradually become a hot topic in the field of PD-L1 research, which will provide new insights to improve the efficacy of current anti-PD-1/PD-L1 therapies. Here, we summarized and discussed multiple PTMs of PD-L1, including glycosylation, ubiquitination, phosphorylation, acetylation and palmitoylation, with a major emphasis on mechanism-based therapeutic strategies (including relevant enzymes and targets that are already in clinical use and that may become drugs in the future). We also summarized the latest research progress of PTMs of PD-L1/PD-1 in regulating immunotherapy. The review provided novel strategies and directions for tumor immunotherapy research based on the PTMs of PD-L1/PD-1.

## 1 Introduction

In the past decade, tumor immunotherapy has emerged as a therapeutic tool and been characterized as the most important scientific breakthrough of the year by *SCIENCE* magazine in 2013 due to its high specificity toward tumor cells and low adverse effects on patients. Immunotherapy mainly stimulates the human immune system to produce tumor-specific immune response in an active or passive way to enhance the immunity of the body against tumor and control/kill tumor cells. At present, five main types of tumor immunotherapy are known: 1) molecular targeted therapy; 2) immune checkpoint inhibitors (PD-1/L1 and CTLA-4 inhibitors); 3) adoptive cellular immunotherapy (CAR-T cellular immunotherapy, TCR-T cellular immunotherapy, etc.); 4) cytokine therapy; 5) tumor vaccines. T cells are activated by recognition of T cell receptor (TCR) peptide-major histocompatibility complexes (MHC) in antigen-presenting cells (APCs) or other target cells and participate in the immune response (1, 2). This process is regulated by a combination of co-stimulatory and co-inhibitory factors involved in the immune checkpoint system. Under normal physiological conditions, the balance between co-stimulatory and co-inhibitory molecules (3) and the balance of immune checkpoint molecules, maintains the optimum immune effect of T cells (4). However, tumor cell growth can disrupt this balance, causing an abnormal upregulation of co-suppressor molecules and their related ligands, such as PD-1 and PD-L1 (5). Pardoll and his co-workers (6) showed that blocking co-inhibitory molecules from binding to ligands (blocking the PD-1/PD-L1 signaling pathway) can reverse the tumor immune microenvironment and enhance and maintain the endogenous anti-tumor effect, resulting in durable tumor control (7). Therefore, immune checkpoint blocker anti-PD-1 and anti-PD-L1 antibodies have now become one of the most promising directions in antitumor therapy (5, 7–9).

Proteins are important performers in the regulation of cellular functions in the organism and affect almost all aspects of normal cell biology and pathogenesis. PTMs are required for proteins to perform their biological functions, and they alter protein stability and activity, are one of the most important modifications in the regulation of protein biological functions. Recent studies on PD-L1/PD-1 have demonstrated that PD-L1 protein levels harbor dynamic changes in the development of the tumor, and corresponding expression changes also occur after immunotherapy, and these dynamic changes are partially regulated by posttranslational modifications (PTMs) (10). Given that PTMs machineries are often therapeutic targets for pharmacological inhibition of cancer, targeting PD-L1 PTMs may be a novel strategy for enhancing antitumor immune responses. Therefore, in this review, we summarized and discussed currently defined multiple PTMs of PD-L1 and the latest research progress of PD-L1/PD-1PTMs in regulating cancer immunotherapy. The review provided references for development of novel strategies and directions for tumor immunotherapy regarding with the PTMs of PD-L1/PD-1.

## 2 PD-L1 and immune escape

PD-L1 (also known as CD274 or B7-H1) serves as the primary ligand for PD-1. The frequency of PD-L1 presence is typically low in the steady state, but it can be expressed in malignant cells, lymphocytes, APCs, hematopoietic cells and epithelial cells in response to certain inflammatory or tumor cell stimuli. In malignant cells, the PD-1/PD-L1 signaling pathway is aberrantly activated, and PD-1 and PD-L1 bind with each other to regulate the proliferation and activity of T cells. This reduces their immune response to surrounding tissues, helping tumor cells achieve immune escape (11, 12). In addition, PD-L1 can protect tumor cells from the cytotoxic effects mediated by interferon and cytotoxic lymphocytes (CTL), even in the absence of PD-1 of T cells (13). Thus, the role of PD-L1 in tumor immunity is remarkably crucial than that of PD-1 because of its characteristics.

The examination of molecular mechanisms of tumor immune escape is one of the core challenges in immuno-oncology research, in which PD-1/PD-L1-mediated immune escape mechanisms are particularly important. PD-1 exerts its effects mainly because of three structural domains in the extracellular, intracytoplasmic and transmembrane parts, which also contain the immunoreceptor tyrosine-based switch motif (ITSM) and the immunoreceptor tyrosine-based inhibitory motif (ITIM) (14). PD-L1 does not have a typical signaling motif because its tail consists of a shorter cytoplasmic group. The interaction of the extracellular structural domain of PD-1 with PD-L1 results in a change in PD-1 conformation and tyrosine phosphorylation in the PD-1 cytoplasmic structural domain, which leads to an increase in the linkage of SHP-2 tyrosine phosphatase to ITSM (15). The increase in SHP-2 leads to a decrease in phosphorylation of TCR molecules. PD-1/PD-L1 inhibitors can block the combination of both, thereby restoring the immune cell-mediated killing of tumor cells (16, 17).

## 3 PTMs of PD-L1 and immunotherapy

Post-translational modification is an important and reversible process for protein regulation. Currently reported PTMs of PD-1/PD-L1 include glycosylation, ubiquitination, phosphorylation, acetylation, palmitoylation. These modifications not only regulate the expression level and stability of PD-L1, but also play an important role in regulating PD-1/PD-L1-related signaling pathways and improving the anti-tumor performance of T cells (18, 19). Therefore, PTMs of PD-1/PD-L1 may emerge as a novel strategy to enhance the efficacy of target PD-1/PD-L1-related drugs.

### 3.1 Glycosylation of PD-L1

Glycosylation modifications are fundamental to the stable expression and normal physiological function of membrane proteins and affect protein activity (20). PD-L1 is inserted into the endoplasmic reticulum to begin the process of glycosylation and is processed and transported through the secretory pathway. The process is completed within the Golgi apparatus. Glycosylated PD-L1 is transferred to the cell membrane to participate in immune regulation of the cell. Once glycosylation is dysregulated, aberrant or non-glycosylated PD-L1 can be recognized by endoplasmic reticulum-associated protein degradation (ERAD) and E3 ligase. This is followed by polyubiquitination, translocation from the endoplasmic reticulum to the cytoplasm and degradation by the proteasome (21). Depending on the glycosidic bond site, glycosylation modifications of proteins can be classified into two types: N-glycosylation and O-glycosylation (22). N-glycosylation is the process that the N-glycan chain is covalently attached to the dissociative NH2 group of the aspartic acid of the protein. O-glycosylation is the process that the O-glycan chain is covalently linked to the dissociative OH group of the serine or threonine of the protein. Glycosylation of proteins usually leads to the observation of heterogeneous patterns on western blot, such as PD-L1 (B45 kDa) (23). After removal of the entire n-glycan structure with recombinant glycosidase (peptide-n -glycosidase F; PNGase F) followed by western blot analysis of cell lysates, it was found that the size of PD-L1 was reduced from 45 kDa to 33 kDa, but O-glycosidase failed to produce a similar effect (24). This indicates that the higher molecular weight PD-L1 is indeed attributed to glycosylated form, which is mainly N-glycosylation. N-glycosylation plays a key role in determining protein structure and function, especially the glycosylation of membrane receptor proteins is important for protein interactions (e.g., between ligands and receptors) and has been shown to affect protein activity (25). N-glycosylation was divided into three subtypes, complex, mixed and mannose-rich, according to the composition of their glycan chains, and the glycosylation type of PD-L1 was mainly complex N-glycosylation. The mass spectrometry analysis showed that the asparagine residues of PD-L1 extracellular structural domains N35, N192, N200, and N219 were highly glycosylated (24). Glycosylation is involved in the stability of PD-L1 structure and PD-1/PD-L1-mediated tumor immunosuppressive function and affects the accuracy of PD-L1 detection.

Three main effects of glycosylation on PD-L1 are known (24): firstly, glycosylation of PD-L1 at the N192, N200 and N219 sites impedes the recognition of PD-L1 binding by E3 ubiquitin ligases, which protects PD-L1 from degradation and enhances its protein stability (24). Secondly, N-glycosylation modification of PD-L1 is fundamental for its binding to PD-1 and its immunosuppressive function. Upon N-glycosylation, it enhances protein stability by blocking phosphorylation and subsequent ubiquitination degradation of the adjacent region of the T180/S184 site (24, 25), which in turn binds to PD-1 and inhibits CLT activity (21, 26). Third, Lee et al. (23), also found that the affinity of glycosylated PD-L1 to PD-L1 antibodies was significantly reduced, which may be due to the fact that deglycosylase PD-L1 may eliminate the gap that exists in the space that would be detected by the antibody. Thus, the use of anti-PD-L1 immunohistochemistry can significantly improve the sensitivity of PD-L1 recognition. Furthermore, the deglycosylation of PD-L1 also has potential acting as a diagnostic biomarker that can well predict the response to PD-L1 immunotherapy and more accurately assess PD-L1 protein levels.

PD- L1 can also express in exosomes as a soluble protein (27). Exosomal PD- L1 exists in a highly N-glycosylated form and plays an important role in the regulation of immune escape. Experiments by Zhu et al, showed that exosomal PD- L1 glycosylation is the key structural basis for PD - L1/PD-1 interaction and inhibition of CD8^+^ T cell proliferation (27). Glycosylated PD- L1 in exosomes may be a promising new target for immunotherapy.

Due to the aforementioned role of glycosylation on PD-L1, Pu et al, proposed the concept of “non-glycosylated PD-L1” for tumor cellular immunotherapy (28). Glycosyltransferase 1 domain-containing 1 (GLT1D1), the staurosporine temperature sensitivity 3 (STT3), G-fructose amidotransferase1 (GFAT1), Glyco-PD-L1-processing enzymes, as well as several molecules and proteins, can be blocked. Non-glycosylated PD-L1 can effectively reduce the interaction between PD-L1 and PD-1, weaken the regulatory effect of tumor cells on T cells, and inhibit the immune escape of tumor cells.

#### 3.1.1 Glycosyltransferase 1 domain 1

GLT1D1 is an enzyme that translocates polysaccharides to target proteins. Current analysis of clinical specimens has demonstrated high expression of GLT1D1 in B-cell non-Hodgkin’s lymphoma and early relapsed diffuse large B-cell lymphoma (29). High expression of GLT1D1 increases PD- L1 glycosylation and promotes tumor immune escape and tumor growth. Hence, it is negatively correlated with patient prognosis. Downregulation of GLT1D1 significantly decreases PD- L1 glycosylation, which leads to a significantly higher proportion of non-glycosylated PD-L1, enhances cytotoxic activity of cytotoxic T cells against lymphoma cells, and influences PD- L1/PD-1 interaction, implying that GLT1D1 can be a novel target for immunosuppressive therapy in non-Hodgkin’s lymphoma (29).

#### 3.1.2 STT3

The ER-associated N-glycosyltransferase STT3 is essential for N-glycosylation and PD- L1 stability. Epithelial-mesenchymal transition (EMT) enriches PD-L1 in CSCs by the EMT/β-catenin/STT3/PD-L1 signaling axis, in which EMT transcriptionally induces N-glycosyltransferase STT3 through β-catenin, and subsequent STT3-dependent PD-L1 N-glycosylation stabilizes and upregulates PD-L1 (30). In hepatocellular carcinoma (HCC), IL- 6 activates JAK1/PD-L1 phosphorylation at the Y112 site, followed by stimulation of STT3A recruitment and subsequent glycosylation to increase PD-L1 expression. Inhibition of the IL- 6/JAK1 pathway resulted in loss of STT3A and subsequent decrease in PD- L1 stability (26). Etoposide (ETO) (30), a DNA topoisomerase inhibitor, inhibits STT3 expression induced by EMT, thereby suppressing STT3-mediated PD-L1 glycosylation modification. More non-glycosylated PD-L1 reduces the stability of PD-L1 proteins in tumor stem cells and promotes the clearance of tumor-infiltrating T lymphocytes (TILs) from tumor stem cells. In addition, ETO improves the therapeutic effect of TIM-3 (hepatitis A virus cellular receptor 2) monoclonal antibody in a tumor-bearing mouse model, further enhancing the CD8+ T cell-mediated anti-tumor immune response. Secondly, in colorectal cancer, KYA1797K inhibits the β-catenin/STT3 signaling pathway and downregulates the expression of STT3, thereby inhibiting PD- L1 glycosylation, reducing its stability and suppressing immune escape (31, 32). In addition, a recent study in nasopharyngeal carcinoma found that transforming growth factor β (TGF-β) promotes upregulation of the expression of glycosyltransferase STT3A via c-Jun, which promotes PD-L1 glycosylation and enhances its stability (33). Therefore, the efficacy of immune checkpoint blockade may be enhanced by interfering with TGF-β or combining it with PD-1/PD-L1 blockade strategies.

#### 3.1.3 G-fructose amidotransferase1

GFAT1 is the rate-limiting enzyme of the hexosamine biosynthetic pathway, generating the glycosylation precursor uridine diphosphate-N-acetyl-glucosamine (UDP-GlcNAc), which is fundamental for the glycosylation of many proteins (34), also one of the substances necessary for the stability of PD-L1 protein. Inhibition of GFAT1 significantly reduces PD-L1 glycosylation and protein stability which leaving more PD- L1 in a non-glycosylated state. And it promotes T cell activity and NK cell antitumor activity, significantly enhancing tumor immunity. This mechanism has been demonstrated in lung cancer (35).

#### 3.1.4 Glyco-PD-L1-processing enzymes

Glyco-PD-L1-processing enzymes are also involved in the regulation of N-linked polysaccharide-mediated PD- L1 modification in tumor cells. Biochemical tests revealed that resveratrol (RSV) (36) can directly inhibit N-linked glycan chain modifying enzymes, retaining unglycosylated PD- L1 in the endoplasmic reticulum, reducing the stability of PD- L1, preventing the migration of fully glycosylated PD- L1 to the cell membrane, and affecting the interaction between extra-membrane PD- L1 and PD- 1, limiting immune escape to a certain extent and significantly enhancing the activity of T lymphocytes. The enhancement of T-cell immune function by RSV mediated by targeting PD-1/PD- L1 immune checkpoints is an important mechanism for its oncogenic function.

#### 3.1.5 Glucose analogues 2-deoxyglucose

2-DG inhibits N-glycosylation modifications. 2-DG, as a glucose analogue with glycolysis inhibitor, significantly inhibits the glycosylation modification of PD-L1 (37). Inhibition of protein n-linked glycosylation using 2-DG enhances anti-tumor T-cell immunity in TNBC *in vivo* (38, 39). Furthermore, co-culture experiments with human peripheral blood mononuclear cells (PBMCs) showed that the combination of 2-DG and Olaparib significantly attenuated the immunosuppressive effect of Olaparib monotherapy on PBMCs and enhanced PBMC-mediated killing of tumor cells (40). 2-DG can play a role in immunotherapy treatment of tumors not only directly but also in combination with other drugs through non-glycosylation, achieving a 1 + 1>2 effect.

#### 3.1.6 β-1,3-N-acetylglucosaminyl transferase

As a type II transmembrane protein in the Golgi apparatus, β-1,3-N-acetylglucosaminyl transferase B3GNT3 (25), is responsible for catalyzing the synthesis reaction of poly-N-acetylgalactose sugar chains and is involved in the glycosylation of various proteins. Li et al, confirmed in cytological experiments that B3GNT3 promotes the glycosylation of PD-L1 in response to EGF stimulation and enhances the binding of PD-L1 to PD-1, thereby inhibiting the killing effect of T cells on tumor cells (25). This effect can be blocked by N-glycosylation inhibitors.

#### 3.1.7 PD-L1-associated chaperone Sigma1

Sigma1 facilitates PD-L1 glycosylation in the endoplasmic reticulum, preventing autophagic degradation of PD-L1, thereby stabilizing PD-L1 in tumor cells. Maher et al, confirmed in their molecular cell experiments that (41) (1-(4-iodophenyl)- 3 -(2-adamantyl) guanidine) (IPAG), an inhibitor of the endoplasmic reticulum molecular chaperone Sigma1, can inhibits the glycosylation modification of PD-L1 and reduces the binding ability of PD-L1 to PD-1, which promotes the killing of tumor cells by CD8^+^ T cells.

#### 3.1.8 Other genes involved in the regulation of glycosylation

In addition, there are other genes involved in the regulation of glycosylation. fK506-binding protein 51 spliceosomes (FKBP51s) act as chaperone molecules for PD-L1 and stabilize PD-L1 in tumor cells by assisting its folding in the endoplasmic reticulum, thereby promoting PD-L1 glycosylation (42). Induction of GSK3β inactivation by EGF in tumor cells leads to increased glycosylation PD-L1. Glycogen synthase kinase 3β (GSK3β) induces phosphorylation-dependent proteasomal degradation of PD-L1 via β-TrCP, but this interaction is antagonized by PD-L1 glycosylation at N192, N200 and N219. Hence, inactivated GSK3β helps increase PD-L1 stability and aids tumor immune escape (24). The Chinese herbal medicine *Shikonin* may inhibit PD-L1 glycosylation through NF-κB and signal transducer and activator of transcription 3 (STAT3), which in turn promotes its degradation (43–45). These enzymes, molecules or their inhibitors can reduce the glycosylation of PD-L1 and therefore may become new targets for immunotherapy (46). The most widely used glycosylation inhibitor tunicamycin (42), however, cannot be used clinically because its own specific structure can affect the glycosylation of many glycoproteins, and the matter that toxin production associated with endoplasmic reticulum stress cannot be resolved.

### 3.2 Ubiquitination of PD-L1

Ubiquitination is the binding of ubiquitin as a monomer or multimer to specific amino acids of proteins with the participation of ubiquitin-activating enzymes. Damaged or unwanted proteins in cells need to be modified by ubiquitination before they can be recognized and degraded by the proteasome (47). The ubiquitin-proteasome pathway is responsible for most of the intracellular protein degradation and involves three enzymes: ubiquitin activating enzymes (E1s), ubiquitin binding enzymes (E2s) and ubiquitin ligases (E3s) The conjugative cascade of the ubiquitin pathway consists of three enzymatic reaction steps (48, 49): first, the ubiquitin C -terminal glycine residue is activated by E1 in an ATP-dependent manner; second, the activated ubiquitin is delivered to the E2 enzyme cysteine residues; third, the E3 enzyme catalyzes the covalent attachment of ubiquitin to the lysine residues of the substrate protein. Ubiquitination and deubiquitination together regulate the half-life of PD-L1. Degradation of PD-L1 by ubiquitination greatly reduces its half-life, decreases its stability, and affects the regulation of binding to PD-1, preventing immune escape of tumor cells. The process of ubiquitination can be reversed by deubiquitination (50, 51).

The different ubiquitin chain modifications of ubiquitination have different functions (52–55), among which the polyubiquitin chains linked by K48 and K11 can be recognized by the 26S proteasome, which in turn degrades the substrate protein. The K63-linked polyubiquitin chain can also be sorted into endosomes by internalization and finally degraded by lysosomes. However, in recent years, strong evidence has shown that PD-L1 protein expression is usually regulated by the ubiquitin (UB)-mediated proteasomal degradation pathway. Therefore, here, we mainly discuss the degradation via the proteasome pathway.

#### 3.2.1 β-TrCP

β-TrCP (also known as BTRC) E3 ubiquitin ligase, often functions in combination with SCF (SKP1-CUL1-F-box protein) to form the SCFβ-TrCP E3 ubiquitin ligase complex. β-TrCP mediates the ubiquitination of proteins involved in cell cycle progression, signal transduction, and transcription in a phosphorylation-dependent manner (56). Phosphorylation of PD-L1 by GSK3β (see Phosphorylation below for details) leads to the association of PD-L1 with the E3 ligase β-TrCP, resulting in the degradation of PD-L1 in the cytoplasm (24). β-TrCP binds to the DSG motif on PD-L1 (where D is aspartate, S is serine, and G is glycine) to catalyze its ubiquitination at the K48 site and subsequent degradation of PD-L1 via the 26S proteasome. Glycosylation of N192, N200 and N219 creates a spatial barrier that disrupts the interaction between GSK3β and PD-L1 (24), leading to stabilization of PD-L1 protein. Inhibition of β-TrCP or certain specific molecules that inactivate GSK3β can in turn block PD-L1 ubiquitination, promote its stability, and ultimately induce cancer immunosuppression. In addition, recent *in vivo* experiments in mice revealed that eukaryotic elongation factor 2 kinase (eEF2K) (57) promotes immunosuppression of melanoma via PD-L1 stabilization mediated by GSK3β inactivation. Screening of the FDA-approved antitumor drug library identified Cytarabine as a potentially clinically applicable eEF2K inhibitor (58). The association revealed that cytarabine could be used synergistically with the downward regulation of the oncogene expression SPP1 by other BET inhibitors (for example I-BET282E) (59) for the treatment of melanoma. This finding was also confirmed in a clinical trial of 38 patients with melanoma treated with anti-PD-1 therapy, providing a potential combination treatment strategy to improve the efficacy of immunotherapy.

#### 3.2.2 SPOP

Speckled POZ protein (SPOP) is a typical CRL3 adaptor protein. SPOP interacts with Cullin scaffold protein 3 in CRL3, and 3 (cullin 3)-SPOP interacts with the 283-290 region of PD-L1 to promote polyubiquitination and degradation of PD-L1 in a cell cycle-dependent manner (49). This process can be catalyzed by the cell cycle protein D-CDK4 (60). Then conversely, SPOP inactivating mutations reduce the ubiquitinated degradation of PD-L1, significantly upregulate PD-L1 expression and reduce the number of tumor-infiltrating lymphocytes at tumor sites. *In vivo* studies have shown that when the MATH structural domain of SPOP polypeptide chain was mutated, the protein content of PD-L1 was significantly increased in the tumor tissues of tumor-bearing mice, improving the therapeutic effect of PD-L1 monoclonal antibody. It is also noteworthy that inhibition of CDK4/6 blocked cell cycle protein cyclin D-CDK4-mediated phosphorylation of SPOP, which significantly increased the expression of PD-L1 protein. Paboxicillin and Ribociclib (61), inhibitors of CDK4/6 combined with PD-L1 antibody enhance the ability of CD8^+^ T cells to secrete IFN-γ (interferon-γ), promote T cell-mediated anti-tumor immune response and significantly increase survival (61).

#### 3.2.3 STUB1

STUB1 ubiquitin ligase is considered a tumor suppressor because it promotes the ubiquitination and degradation of some oncogenic proteins. It also negatively regulates the suppressive activity of regulatory T cells (Treg) by promoting the degradation of the transcription factor Foxp3, which is often reduced or absent in cancer cells (62, 63). Similarly, STUB1 ubiquitin ligase catalyzes the ubiquitination of lysine sites in the cytoplasmic region of PD-L1, promoting PD-L1 degradation in a proteasome-dependent manner and is involved in regulating PD-L1 stability. The knockdown of CMTM6 induces polyubiquitination of PD-L1 in circulating endosomes, which leads to degradation via the lysosomal pathway (53, 64). In addition, Mezzadra et al. (53), and Burr et al. (64), showed that the binding of CMTM6 to PD-L1 mediates the internalization of a larger fraction of PD-L1 on the cell membrane and recirculation to the plasma membrane and circulating endosomes. Furthermore, CMTM6 also specifically regulates PD-L1 in these two cell compartments. This results in reduced ubiquitination of PD-L1 during the cell cycle and lysosomal degradation of PD-L1 during the cell cycle, thereby prolonging its half-life, inducing and stabilizing PD-L1 expression at the cell membrane, and enhancing the ability of PD-L1-expressing tumor cells to suppress T cells and cancer cells for evasion of immune surveillance. Notably, STUB1 downregulation leads to a significant upregulation of PD-L1 expression in CMTM6-insufficient cells compared with that in CMTM6-proficient cells, which suggests that STUB1 initiates the ubiquitination of PD-L1, either indirectly or through direct regulation of the lysine in the PD-L1 cytoplasmic domain.

#### 3.2.4 HMG-coenzyme A reductase degradation protein 1

In the endoplasmic reticulum, the E3 ubiquitin ligase HRD1 is involved in the immune regulation of antigen-presentation function of dendritic cells and sensitization of T and B lymphocytes. Deletion of the HRD1 gene results in a corresponding decrease in the number of T cells, and the clonal expansion and differentiation of T cells is inhibited. Therefore, HRD1 is considered to be a positive phase regulator of T cell activity. HRD1 induces polyubiquitination of aberrantly glycosylated PD-L1, leading to degradation of PD-L1 via the ERAD pathway (65). Reduction of HRD1 significantly blocked metformin-stimulated ubiquitination of endogenous PD-L1 and revealed that HRD1 acts as a positive regulator of T-cell activity in immune regulation (21).

#### 3.2.5 Defective cullin neddylation 1 domain‐containing 1

Defective cullin neddylation 1 domain‐containing 1/squamous cell carcinoma‐related oncogene (DCUN1D1/SCCRO) is a ring-finger domain ubiquitin E3 enzyme that is involved in the growth and metastasis of malignancies such as colorectal (66), glioma (67) and prostate cancers (68). A recent study revealed the oncogenic mechanism of DCUN1D1 in non-small cell lung cancer (69). Upregulation of the expression of DCUN1D1 significantly increased the levels of PD - L1 protein in non-small cell lung cancer cells. The regulatory mechanism may be related to FAK pathway; however, the exact mechanism of action is not fully understood.

#### 3.2.6 Other enzymes and factors involved in the regulation of ubiquitination

Additionally, there are several enzymes and factors involved in the regulation of ubiquitination of PD-L1. Metformin-stimulated amp-activated protein kinase (AMPK) phosphorylates PD-L1 at the S195 site, blocking its ER-to-Golgi translocation and leading to the degradation of PD-L1 by the ERAD system (21). E3 ligase Neural precursor cell express developmental downregulated protein 4 (NEDD4) can be inhibited by activated fibroblast growth factor receptor 3 (FGFR3) in tumor cells, contributing to polyubiquitination and degradation of PD-L1 (70). The membrane-bound ubiquitin ligase RNF144A interacts with PD-L1 in the cytosolic membrane and intracellular vesicles to promote polyubiquitination and proteasomal degradation of PD-L1 (71). Epidermal growth factor (EGF) (72) induces PD-L1 monoubiquitination and increases protein expression. In addition, epidermal growth factor receptor (EGFR) inhibitors Osimertinib significantly reduce PD-L1 ubiquitination (73, 74). The Cbl family members, c-Cbl and Cbl-b, are RING finger E3 enzymes that catalyze the transfer of ubiquitin from specific E2 enzymes to target substrates. They can inhibit PD-L1 expression by inactivating STAT, AKT, and ERK signaling pathways and are also promising therapeutic manipulation targets for anti-PD-1/PD-L1 tumor immunotherapy. CDK5 can mediate activation by phosphorylation of FBXO22, which acts as E3 to promote degradation of PD-L1 in lung cancer cells (75). A recent study identified ARIH1 as an E3 ubiquitin ligase responsible for targeting PD - L1 degradation associated with GSK3α phosphorylation (see below for details of the mechanism involved) and promoting antitumor immunity, suggesting that ARIH1 may be a potential drug target for enhancing antitumor immunity and immunotherapy (76).

A growing body of data shows that, blocking the interaction between PD-1 and PD-L1 by anti-PD-1/PD-L1 monoclonal antibody has shown great anti-tumor efficacy in various kinds of solid tumors (77). However, many immune related adverse reactions with fatal consequences of monoclonal antibodies have been reported in these years. Based on this situation, small molecule immunotherapy has emerged. PROTACs are ternary chemical complexes that usually consist of three functional parts, an E3 ligase-recruiting chemical ligand, a POI-binding chemical ligand and a linker (78, 79). It uses ubiquitination mechanism to degrade the target protein via both proteasomal and lysosomal pathways to achieve the inhibition of the target protein, and enable targeted degradation of proteins hard to target by conventional methods. We have mentioned above that PD-L1 protein is subject to ubiquitin-mediated proteasomal degradation, therefore, it is feasible to design novel PD-L1 small molecule degraders based on PROTAC technology. Typically, von Hippel–Lindau disease tumor suppressor (VHL) and Cereblon (CRBN) are the most commonly used endogenous E3 ligases in the PROTAC field. Cheng et al. (80), synthesized a novel resorcinol diphenyl ether-based PROTAC molecule P22 for the first time, targeting the involvement of PD-1/PD-L1 pathway. Not only did it inhibit PD-1/PD-L1 interaction, but also moderately reduced PD-L1 protein levels in a lysosome-dependent manner, enhancing the anti-tumor effect of PD-L1 antibody. Liu et al, designed a group of PROTACs consisting of multiple PD-L1 extracellular segment ligands (BMS-37) -junction -CRBN ligands in three parts. The most active PROTAC molecule, BMS-37-C3, has been confirmed to significantly enhance the killing ability of T cells while reducing the protein level of PD-L1 in various molecular tests (81, 82). Besides the traditional inhibitor based PROTAC of PD-L1, Cotton et al, developed first antibody-based PROTACs (AbTACs) inducing the degradation of PD-L1, which can target both PD-L1 and the E3 ligase RNF-43 to induce the lysosomal degradation of PD-L1 (83).

### 3.3 Deubiquitination of PD-L1

As mentioned above, ubiquitination is an essential protein post-translational modification process that degrades proteins via the proteasomal pathway, thereby affecting various physiological metabolisms within the cell. Deubiquitination is the reverse process of ubiquitination modification, and this process requires the involvement of deubiquitinase (DUB). DUB reverse regulates the ubiquitination process by removing individual ubiquitin molecules or polyubiquitin chains from the tagged target protein by hydrolyzing the peptide bond at the carboxyl terminus of ubiquitin, ester bond or isopeptide bond. The reversible regulation of protein ubiquitination modification and deubiquitination puts the protein expression level in dynamic equilibrium, maintains the stability of its expression level and function, and affects the function of proteins in cellular life activities.

#### 3.3.1 COP9 signalosome 5

The constitutive COP9 signalosome 5 acts as a large multiprotein complex, similar to the 19S lid of the 26S proteasome and plays an integral role in the regulation of cullin-RING ubiquitin E3 ligases (CRLs). CSN5 is the fifth member of the CSN family and is involved in a subgroup of biological processes that including transcription factor specificity, denuclearization modification of NEDD8 and nuclear to cytoplasmic translocation of primary molecules. CSN5 is associated with cancer survival and is considered a biomarker of poor prognosis in some tumors. Consistently, CSN5 acts as a DUB with deubiquitinating activity and is a negative regulator of PD-L1 ubiquitination (52, 84). Macrophages secrete the pro-inflammatory cytokine TNF-α to activate NF-κB and induce tumor cells to express CSN5 (85), which subsequently inhibits ubiquitination and degradation of PD-L1, thereby enhancing PD-L1/PD-1 interactions and evading immune surveillance by T cells. However, the CSN5 inhibitor curcumin reversed this situation and improved the therapeutic efficacy of CTLA4 blockade therapy (52). CC chemokine receptor 5 (CCR5) and its ligand ligands (e. g. CCL5) are involved in the suppressive effect of tumor-associated macrophages (TAMs), one of the most potent immune cell types in the cancer tumor microenvironment, on CD8^+^ T cell immunity. They demonstrate oncogenic and immunosuppressive effects. The activity is further enhanced by the production of NF-kB p65/signal transducer and activator of transcription 3 (STAT3) complexes linked to the CSN5 promoter by macrophage-derived CCL5 (85). The NF-KB inhibitor Shikonin (46) decreased PD-L1 glycosylation and increased PD-L1 degradation, whereas activated STAT3 and overexpressed CSN5 reversed these trends. Thus, stabilization of PD-L1 by inhibiting NF-kB/CSN5 is a potential strategy to treat cancer-associated inflammation. Overall, CSN5 plays an important role in PD-L1 regulation and may be a promising therapeutic target in tumor immunotherapy.

#### 3.3.2 Ubiquitin-specific proteases

A variety of USPs are involved in the regulation of PD-L1 deubiquitination mediated by different mechanisms. USP22 is observed in a variety of malignancies and is especially highly expressed in HCC. It interacts with the C-terminus of PD-L1, deubiquitinates PD-L1 (86), and inhibits its degradation via the USP22-CSN5-PD-L1 axis (87), which is closely associated with the prognosis of HCC (88). Moreover, in NSCIC (86), USP22 deletion can promote the therapeutic effect of PD-L1-targeted tumor immunotherapy. Recent experiments in mouse models of lung cancer have shown that (89) targeting USP7 with USP7 inhibitor P5091 upregulates the expression of PD-L1 protein in Lewis tumor cells and blocks PD-1, leading to an effective anti-tumor response in lung cancer. However, the underlying mechanism of its inhibition of PD-L1 upregulation is still unclear. Another ubiquitin-specific protease, USP9X (also known as FAM), which reduces PD-1 expression on T cells is involved in immune regulation of tumors. As a member of DUBs, ubiquitin-specific peptidase 9, Xlinked (USP9X) has a role in the control of tumor cell proliferation, adhesion, and apoptosis, among other things (90). And it has been found to be inappropriately expressed in non-small cell lung cancer, melanoma as well as breast cancer (91–93). Next, USP9X was found to be expressed at high levels in EGR-positive prostate cancer and the USP9X inhibitor WP1130 was found to induce ERG degradation and thus inhibit tumor growth (94). In addition, USP9X is highly expressed in oral squamous cells carcinoma (OSCC) cells. The high expression of USP9X in OSCC cells increases the stability of PD-L1 in OSCC cells by deubiquitinating the tumor and promoting immune escape (95). Therefore, targeting PD-1/PD-L1 by inhibiting the activity of USP9X may be a promising anti-cancer therapeutic strategy. In addition, USP21 (96), a novel deubiquitinating enzyme of PD-L1, was recently identified. Its overexpression significantly upregulated PD-L1 expression.

#### 3.3.3 OTU domain, ubiquitin aldehyde binding 1

The deubiquitinating enzyme OTUD1 is involved in the deubiquitination of apoptosis inducing factor (AIF) and plays a role in regulating apoptosis. It interacts with the K48-linked polyubiquitin chain in the intracellular region of PD-L1 and impedes the degradation of PD-L1 via the ERAD pathway by mediating its deubiquitinase activity (97). In non-small cell lung cancer experiments, the overexpression of the cyclic RNA insulin-like growth factor 2 mRNA-binding protein 3 (circIGF2BP3) upregulates the expression of OTUD1 by stabilizing mRNA (98). This, in turn, antagonizes the ubiquitinated degradation of PD-L1 and suppresses CD8^+^ T cell function, leading to immune escape. In addition, in mice with breast cancer (97), the number of infiltrating CD8^+^ T cells and the serum levels of IFN-γ were significantly increased after PD-L1 destabilization induced by OTUD1 deletion, promoting tumor immunotherapy.

### 3.4 Phosphorylation of PD-L1

Phosphorylation modification is the process of transferring ATP phosphate groups to amino acid (tyrosine, serine, threonine) residues of substrate proteins catalyzed by protein kinases. The phosphorylation sites of PD-L1 are mainly concentrated in the extracellular structural domain and are often found together with the sites of glycosylation and ubiquitination (24). However, different kinases induce phosphorylation of different sites of PD-L1 with completely different effects. Among the five widely studied protein kinases that mediate PD-L1 phosphorylation, GSK3β/α and AMPK-mediated phosphorylation of PD-L1 leads to degradation by ubiquitination in the cytoplasm, whereas JAK1 and NIMA-associated kinase 2 (NEK2)-mediated phosphorylation of PD-L1 stabilizes PD-L1 by promoting PD-L1 glycosylation and inhibiting PD-L1 ubiquitination.

#### 3.4.1 Glycogen synthase kinase 3β/α

GSK3β induces phosphorylation of non-glycosylated PD-L1 at the T180 and S184 sites of its extracellular structural domain by binding to the post-translational motif of PD-L1 (S/TXXXS/T, where S is serine, T is threonine and X is any amino acid) (24). The phosphorylated PD-L1 is further ubiquitinated by K48 via binding to the E3 ligase β-TrCP. This induces degradation of PD-L1 via polyubiquitination in the cytoplasm. For PD-L1 that has undergone glycosylation, the glycosylation of N192, N200 and N219 blocks its spatial site, inhibiting the effect of GSK3β on PD-L1 and preventing PD-L1 phosphorylation and subsequent degradation. Here, β-TrCP mediates the GSK3β-dependent phosphorylation of PD-L1 for PD-L1 degradation. In contrast, GSK3β-independent PD-L1 needs to be induced by mTORC1/p70S6K to phosphorylate it, which, in turn, leads to β-TrCP-mediated PD-L1 degradation (99). PARP1 inhibitor Olaparib (100, 101), tyrosine kinase inhibitors (TKIs) (100), and resveratrol (36) inhibit GSK3β activity, which further affects the interaction between PD-L1 and β-TrCP. In addition, epidermal growth factor (EGF) acts as an upstream signal that regulates the expression and function of GSK3β protein. Gefitinib, Erlotinib, Osimertinib and ES-072 are inhibitors of the EGF receptor EGFR (24, 76, 102, 103), induce phosphorylation of non-glycosylated PD-L1, rendering it susceptible to degradation. Secondly, the novel apropionate derivative SA-49 (104) can enhance the killing of co-cultured tumor cells by NK cells and T cells via the PKCα-GSK3β cascade, which ultimately promotes PD-L1 lysosomal degradation.

In addition, GSK3α also plays a role similar to that of GSK3β (24). GSK3α phosphorylates the S279 and S283 sites of PD-L1, and the phosphorylated PD-L1 is then degraded by the proteasome mediated by the E3 ubiquitin ligase ARIH1 mentioned above (76). Therefore, GSK3α/GSK3β may be a potential target for regulating PD-L1 phosphorylation/ubiquitination.

#### 3.4.2 Metformin activates the AMP-activated protein kinase

AMPK is a key molecule that stimulates glucose utilization by phosphorylating targets involved in glucose transporter transport. It is an enzyme necessary for the body to maintain glucose homeostasis. AMPK phosphorylation is also involved in the regulation of PD-L1, and although it phosphorylates different sites of PD-L1 to promote PD-L1 degradation, the mechanisms are not the same. First, phosphorylation of PD-L1 at the S195 site (21) leads to abnormal PD-L1 glycosylation. The aberrantly glycosylated PD-L1 cannot be transferred from the endoplasmic reticulum to the Golgi apparatus, resulting in a large amount of aberrantly glycosylated PD-L1 being degraded via the ERAD pathway of endoplasmic reticulum-associated degradation. The study confirmed that D-mannose (105) can activate AMPK, which is involved in regulating the whole process mentioned above. Combined treatment with D-mannose and PD-1 blockade therapy in mice greatly inhibited the growth of TNBC and prolonged the survival of tumor-bearing mice. Metformin (106), an AMPK agonist, induced PD-L1 degradation via the ERAD pathway (105) by activating its serine protein kinase activity. It is a process that inhibits the immune escape of tumor cells by reducing the stability and membrane localization of PD-L1. Second, because abnormal energy status leads to cancer development, and energy deprivation activates AMPK, the activated AMPK mediates PD-L1 phosphorylation at the S283 site (107), which disrupts its interaction with chemokine superfamily member 4 (CMTM4) and leads to PD-L1 degradation. In addition, Cha et al. (108), demonstrated that metformin significantly improved the therapeutic effect of immune checkpoint CTLA-4 (cytotoxic T-lymphocyte associated protein 4) monoclonal antibody in mice. The combination of these two drugs promoted the secretion of granzyme B (GZMB) by CD8^+^ T cells and enhanced the killing effect of TILs cells in breast cancer cells. These findings indicate a novel hypothesis for cancer immunotherapy.

#### 3.4.3 Tyrosine kinase JAK1

In 2019, Chan et al, demonstrated that the tyrosine kinase JAK1 induces phosphorylation of PD-L1 at the Y112 site upon interleukin-6 (IL-6) stimulation (26). JAK1 binds to PD-L1 in the endoplasmic reticulum and promotes phosphorylation at the PD-L1 Y112 site, thereby recruiting oligosaccharyltransferase (OST). OST acts on the same position of the n-glycosyltransferase STT3A to catalyze PD-L1 glycosylation and upregulate PD-L1 expression, thereby promoting PD-L1 stability and inducing tumor immune escape. This has been demonstrated by the results of *in vitro* CD8^+^ T cell killing experiments (26), where the protein stability of PD-L1 in cancer cells was reduced when a point mutation of Y112 was noted in PD-L1, rendering PD-L1 unable to bind to PD-1 on the membrane surface of CD8+ T cells. This, enhanced the killing effect of CD8^+^ T cells on tumor cells. Phosphorylation of PD-L1 and its stability is closely related to its stability and may also serve as a potential target for enhanced immunotherapy. There are already drugs based on JAK1 such as to Tofacitinib, Ruxolitinib and Fedratinib (76, 109, 110). And the combination of IL-6 inhibitor with anti-T cell immunoglobulin mucin-3 (anti-Tim-3) enhances the efficacy of T cell-mediated killing of tumor cells (26, 76).

#### 3.4.4 NIMA related kinase 2

In a study of cytological experiments in pancreatic cancer, Zhang et al (111), confirmed that dephosphorylation of PD-L1 by NEK2 is one of the main reasons for the poor immunotherapeutic effect observed with pancreatic cancer. NEK-binding motifs (F/LXXS/T) could be identified in the glycosylation-rich region of PD-L1 (112). NEK2 interacts with PD-L1, phosphorylates T194/T210 residues and prevents ubiquitin-proteasome pathway-mediated degradation of PD-L1 in the ER lumen. This is followed by further promotion of glycosylation at the N192, N200 and N219 sites, preventing PD-L1 degradation and promoting PD-L1 stability (111). Hence, we can speculate that the inhibition of NEK2 and PD-L1 is a promising anti-cancer strategy. And a small molecule drug NCL 00017509, based on NEK2, is currently in preclinical studies (111).

In addition, phosphorylation of heat shock transcription factor 1 (HSF1) (113), a major regulator of the proteotoxic stress response, at the Thr120 site induces its binding to the PD-L1 promoter and upregulates PD-L1 expression. It is expected to be a new immune target.

### 3.5 Acetylation of PD-L1

Acetylation is the process of adding acetyl groups to protein residues, which is mediated by acetyltransferases of acetyl coenzymes A (114, 115) [including histone acetyltransferases (HATs), lysine acetyltransferases (KATs), and Nα-acetyltransferases (NATs) (116)]. And this process can be reversed by deacetylases of acetylated proteins [including histone deacetylases (HDACs) and Sirtuins (SIRTs) (117)]. PD-L1 acetylation can promote tumor immune escape along with glycosylation, but they show completely different mechanisms. As mentioned above, PD-L1 glycosylation inhibits its ubiquitination-mediated degradation, prolongs the half-life of PD-L1, maintains PD-L1 stability, and promotes its binding to PD-1 to help tumor immune escape. Differently, PD-L1 acetylation is able to translocate PD-L1, which is mainly expressed on the cell membrane to function, into the nucleus (118). The accumulation of PD-L1 in the nucleus helps tumor cells evade immune surveillance during metastasis and promotes further tumor development and metastasis.

#### 3.5.1 Histone acetyltransferase P300

PD-L1 is acetylated by p300 at the Lys263 site in the cytoplasmic structural domain, promoting the translocation of PD-L1 to the nucleus via cytocytosis and nucleoplasmic translocation pathways (118). The increased levels of PD-L1 in the nucleus lead to its bindings to DNA and is involved in the regulation of IFN, nuclear factor κB (NF-κB), major histocompatibility complex I (MHCI), and other immune response genes, thus promoting tumor immune escape. HDAC2 inhibitors Trichostatin A in combination with anti-PD-1 antibodies enhance tumor growth inhibition and improve survival in MC38 homozygous use models (119, 120). Therefore, inhibition of HDAC2 in combination with PD-1/PD-L1 blockade is a new strategy for tumor immunotherapy. In addition, binding of hepatitis B virus X-interacting protein (HBXIP) to P300 in breast cancer enhances acetylation of the PD-L1 K270 site, leading to stabilization and accumulation of PD-L1 in cancer cells, thereby enhancing tumor immune escape (121). A study by Gao et al. (118), found that HDAC2 genetic depletion or pharmacologic inhibition of HDAC2 reduced the nuclear portion of PD-L1, thereby enhancing the antitumor efficacy of PD-1 blockers.

#### 3.5.2 Huntingtin interacting protein 1-related

After deacetylation of PD-L1 on the cell membrane by unacetylated PD-L1 or by HDAC2, HIP1R binds specifically to the C-terminus of PD-L1, allowing the β-subunit (AP2B1) of the lattice protein-dependent endocytic junction protein complex (AP2) to recognize HIP1R via a double leucine motif D/E-x-xx-x-L-L/I and bind to PD-L1 to form a complex. The formation of this complex is mediated by lattice proteins to achieve endocytosis, accumulation in the nucleus, and enhancement of the activation of multiple immune response pathways (122). PD-L1 acetylation can mediate its translocation and degradation, but its role in these processes requires further investigation. High expression of PD-L1 is widely used as a marker for patient selection, and we also speculate PD-L1 nuclear expression or PD- L1 acetylation status could be a useful biomarker for future cancer immunotherapy.

### 3.6 S-palmitoylation of PD-L1

Protein palmitoylation, also known as S-palmitoylation, is a reversible form of PTM of protein lipidation in which palmitoyl groups are attached to the sulfhydryl groups of cysteine residues of proteins by thioester bonds. This process is usually catalyzed by a family of DHHC protein acyltransferases (DHHC-PATs) containing the Asp-His-His-Cys active center (123, 124). S-palmitoylation mainly affects protein membrane anchoring, transport, and degradation.

#### 3.6.1 Aspartate-histidine-histidine-cysteine rich sequence

Yang et al. (125), first reported that palmitoyl transferases can interact with PD-L1 in breast cancer and catalyze its palmitoylation at the Cys272 site. Yao et al (126, 127), recently reported a similar finding in a mouse colon cancer model. C272 is the main site of palmitoylation, and after palmitoylation of PD-L1 at the C272 site in the cytoplasm, PD-L1 inhibits the ubiquitination of PD-L1 and prevents its movement to the multivesicular body (MVB). This prevents PD-L1 from being degraded by the lysosomal degradation pathway, thus inducing the development of tumor immune escape. The mutated C272 site inhibits PD-L1 palmitoylation, reduces PD-L1 levels on the membrane surface and its binding to PD-1, and continuously activates T cell-mediated cytotoxicity. Bromohexadecanoic acid (2-bromopalmitate, 2-BP) (126), as an inhibitor of DHHC-PATs enzyme activity, reduces the protein stability of PD-L1 by inhibiting its palmitoylation modification and is the only validated effective PD-L1 palmitoylation targeting drug. However, the inhibitory effect of 2-BP on DHHC-PATs is not specific (127). In the subsequent studies, the development of targeted drugs that specifically inhibit DHHC3/9 could be considered to achieve specific modulation of PD-L1 palmitoylation, and targeting PD-L1 palmitoylation could increase the sensitivity of tumor cells toward T-cell-mediated killing and retard tumor growth. Next, the investigators designed a PD-PALM peptide based on the amino acid sequence profile near the C272 site. It inhibits the palmitoylation of endogenous PD-L1 by competitively binding DHHC3 enzyme, reduced the level of PDL1 expression in tumor cells, and enhanced T cell-mediated anti-tumor immune responses. Clinical studies (114) also found PD-L1 palmitoylation in cisplatin-resistant bladder cancer cells, and inhibition of fatty acid synthase (FASN) inhibited PD-L1 palmitoylation and its expression. The PTMs of PD-L1 and immunotherapy summarized above are detailed in **Table 1**.

**Table 1:** Various PTMs of PD-L1.

	Related enzymes	Modification site	Biological effects	Cancer type	Molecules	References
**N-glycosylation**	GLT1D1	N35 /N192 /N200 /N219	Enhanced stability of PD-L1	Non-Hodgkin’s		(29)
	STT3	N35 /N192 /N200 /N219	Enhanced stability of PD-L1	Liver cell carcinoma	IL- 6	(30)
				Colon cancer		(31, 32)
				Nasopharyngeal Carcinoma	TGF-β	(33)
	GFAT1	N35 /N192 /N200 /N219	Enhanced stability of PD-L1	Lung cancer		(34)
	Glyco-PD-L1-processing enzymes		Reduced stability of PD- L1; Blocking PD-L1 from binding to PD- 1 binding	Breast cancer		(36)
	2-DG		Blocking PD-L1 from binding to PD- 1 binding	Triple negative breast cancer		(37–40)
	B3GNT3	N192 /N200	Promoting PD-L1 binding to PD-1		EGF	(25)
	Sigma1		Enhanced stability of PD-L1	Prostate Cancer/Triple negative breast cancer		(41)
	FKBP51s		Enhanced stability of PD-L1	Glioma	GSK3β,b-TrCP	(42)
	STAT3		Suppresses glycosylation of PD- L1, Activates the NF-kB/STAT3 and NF-kB	Pancreatic cancer		(43–46)
**Ubiquitination**	β-TrCP	K48	Catalytic degradation of PD-L1	Breast cancer	GSK3β, mTORC1/ p70S6K	(56, 57)
	SPOP		Decreases PD-L1 level	Prostate cancer	Cullin 3, D-CDK4/6	(60, 61)
	STUB1		Downregulates level of PD-L1	Melanoma	CMTM6	(53, 62–64)
	HRD1		Downregulates level of PD-L1, Positively regulates T‐cell immunity	Breast cancer	ERAD, Metformin	(65)
	DCU N1D1		Increases PD-L1 level	Colorectal cancer, Glioma, Prostate cancer and Lung cancer	FAK Pathway	(68, 69)
	NEDD4	K48	Promotes PD-L1 degradation	Bladder caner	FGFR3	(70)
	RNF 144A		Promotes PD-L1 degradation	Bladder Tumor	EGFR	(71)
	c-Cbl Cbl-b		Inhibition of PD-L1 expression	Melanoma, Gastric cancer, NSCLC	STAT5a, AKT, and ERK signaling pathways	(73, 74)
	ARIH1		Degradation of PD - L1	Breast cancer	GSK3α	(76)
**Deubiquitination**	CSN5		suppresses degradation of PD-L1	Triple negative breast cancer, NSCLC	TNF-α, NF-κB signaling pathway	(52, 84)
	USP22		Enhanced stability of PD-L1	Liver Cancer, NSCLC, PDA	CSN5	(86–88)
	USP7		Enhanced stability of PD-L1	Lewis lung carcinoma	FOXP3	(89)
	USP9X		Enhanced stability of PD-L1	OSCC, prostate cancer	EGR	(94, 95, 128)
	OTUD1	K48	Blocking PD-L1 degradation	Triple negative breast cancer	ERAD, circIGF2BP3	(97, 98)
**Phosphorylation**	GSK3β	T180/S184;	Promotes β-TrCP-mediated PD-L1 degradation	Gastrointestinal tumors, Breast cancer, Lung cancer, Renal cell carcinoma, etc.	β-TrCP, EGF, EGFR	(24, 99, 100, 104)
	GSK3α	S279/S283	Promotes ubiquitinated degradation of PD-L1	Colon cancer, Cervical cancer, Pancreatic cancer, Lung cancer, Prostate cancer	ARIH1	(24, 76)
	JAK1	Y112				(26, 76)
	AMPK	S195	Aberrant glycosylation of PD-L1 upon degradation by ubiquitination	Breast cancer	ERAD, D-mannose	(21, 105, 106)
		S283	Induction of PD-L1 degradation	Breast cancer	metformin, CMTM4	(107)
	NEK2	T194/T210	Glycosylation of PD-L1 to promote its stability	Liver Cancer	IL-6, OST, STT3A	(111, 112)
**Acetylation**	P300	K263/K270	Promotes PD-L1 internal transfer	Liver cell carcinoma	NF-κB, MHCI, HDAC2	(118, 119, 121)
	HIP1R		Promotes PD-L1 internal transfer	AP2B1		
**S-palmitoylation**	DHHC	C272	Colon cancer, Breast cancer, Bladder cancer	Protects PD-L1 from degradation by lysosomes		(126, 127)

## 4 Interactions and correlations of PTMs of PD-L1

In the regulation of different post-translational modifications of PD-L1, they do not work singularly, but rather are closely related and participate in the regulation of PD-L1 in collaboration.

**Figure 1:**
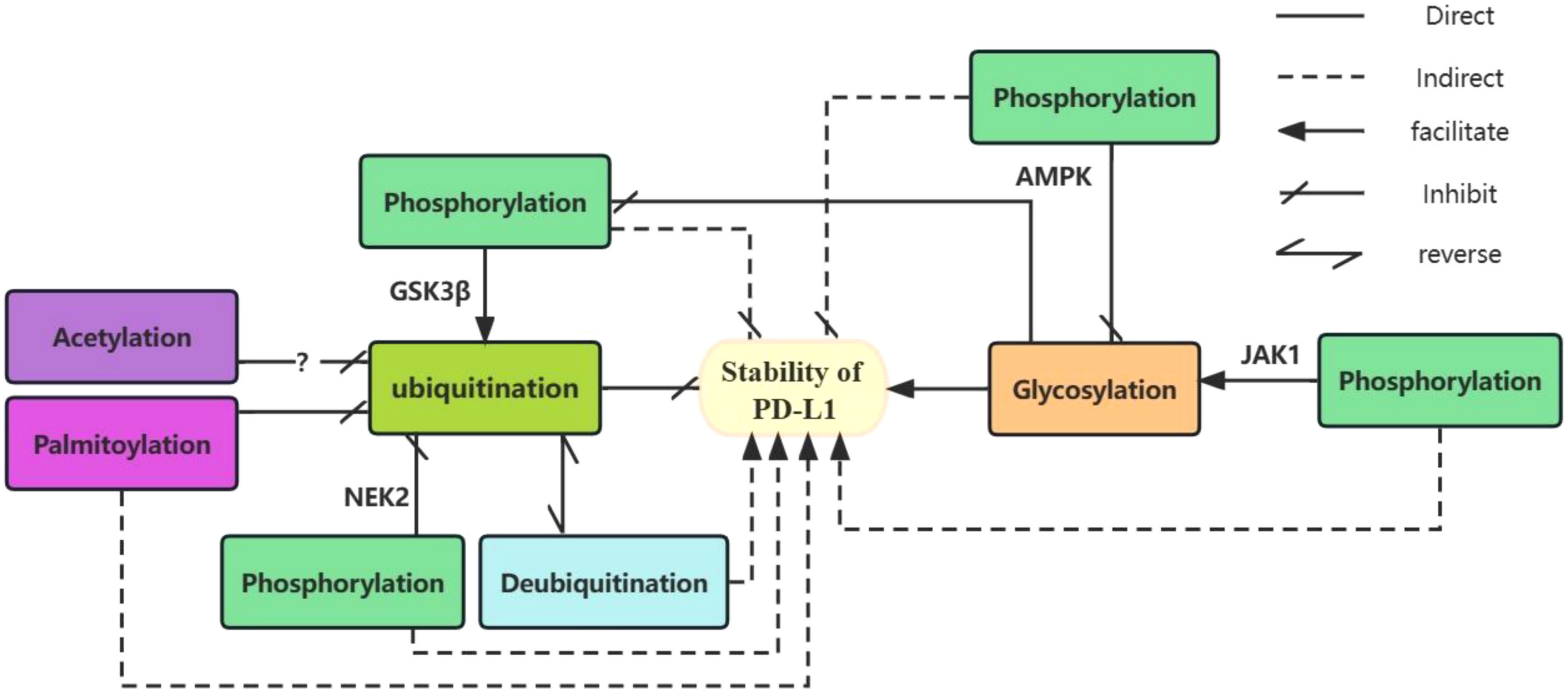
The relationship between several PTMs of PD-L1. Glycosylation and ubiquitination of PD-L1 play a direct role in promoting and inhibiting its stability. Deubiquitination indirectly promotes the stability of PD-L1 by antagonizing its ubiquitination. Different enzyme-mediated phosphorylation of PD-L1 plays different roles. GSK3β-mediated phosphorylation which can be inhibited by glycosylation promotes ubiquitination of unglycosylated PD-L1 and indirectly inhibits its stability. AMPK-mediated phosphorylation inhibits glycosylation of PD-L1 and indirectly reduces its stability. In contrast, JAK1-mediated phosphorylation facilitates PD-L1 glycosylation, indirectly contributing to its stability. Both NEK2-mediated phosphorylation and palmitoylation indirectly promote PD-L1 stability by inhibiting ubiquitination of PD-L1. Acetylation of PD-L1 may inhibits its ubiquitination.

Glycosylation of PD-L1 is involved in the regulation of its ubiquitination and phosphorylation. GSK3β-induced phosphorylation of non-glycosylated PD-L1 activates β-TrCP-mediated degradation of PD-L1 (99). Glycosylation antagonizes the binding of GSK3β to PD-L1 and inhibits PD-L1 phosphorylation (24). Thus, glycosylation of PD-L1 can directly inhibit its phosphorylation and indirectly inhibit its ubiquitination and degradation. PD-L1 glycosylation does not affect its acetylation and nuclear translocation. AMPK phosphorylates PD-L1 at the Ser195 site leads to aberrant glycosylation of PD-L1 and impaired translocation from the endoplasmic reticulum to the Golgi apparatus, resulting in the accumulation of PD-L1 in the endoplasmic reticulum and promoting degradation via. ubiquitination (21). With IL-6 stimulation, JAK1 binds to PD-L1 in the endoplasmic reticulum and promotes phosphorylation at the PD-L1 Y112 site, lead to the n-glycosyltransferase STT3 to catalyze PD-L1 glycosylation and upregulate PD-L1 expression, thereby promoting PD-L1 stability (26). NEK2 (111) induced phosphorylation of PD-L1 inhibits PD-L1 ubiquitination, thereby increasing PD-L1 stability. S-palmitoylation of PD-L1 at the C272 site in the cytoplasm, PD-L1 inhibits the ubiquitination of PD-L1 and prevents its movement to the multivesicular body (MVB) (116, 117). Peracetylation of forkhead box P3(Foxp3), has been reported to inhibit its ubiquitination and degradation (129). And Lysine acetylation of non-histone proteins can compete with ubiquitination and thus affect the stability or subcellular localization of the protein (115). However, there is no clear support for whether PD-L1 acetylation will prevent its ubiquitination in cancer cells and further studies are needed. (See **Figure 1** for details of the relationship between several PTMs of PD-L1).

Glycosylation-targeted drugs prevent immune escape of tumor cells by inhibiting glycosylation and increasing the number and proportion of non-glycosylated PD-L1. Ubiquitination-targeted drugs mainly promote the process of PD-L1 ubiquitination to exert their anti-tumor effects. Phosphorylation-targeted drugs exert their anti-tumor effects mainly by regulating related kinases to achieve the effect of inhibiting glycosylation or promoting ubiquitination degradation. Acetylation-targeted drugs inhibit PD-L1 acetylation and prevent its translocation into the nucleus, preventing immune cells from evading immune surveillance to exert their antitumor effects. Palmitoylation-targeted drugs promote palmitoylation, prevent PD-L1 glycosylation, and reduce PD-L1 stability (See **Table 2** for details of specific drugs and mechanisms of action).

**Table 2:** PTMs of PD-L1 currently exist for clinical treatment.

	Drug name	Combination therapy	Target	Action mechanism	References
**N-Glycosylation**	Etoposide	Etoposide + TIM-3 mAb	STT3	Inhibiting EMT-induced STT3 expression and reducing PD-L1 stability	(30)
	2-DG	2-DG + Olaparib	2-DG	Inhibition of PD-L1 Glycosylation Modification	(40)
	IPAG	Unknow	Sigma1	Inhibition of PD-L1 Glycosylation Modification	(41)
	*Shikonin*	Unknow	STAT3	Inhibition of PD-L1 Glycosylation Modification	(43–46)
**Ubiquitination**	Resveratrol	Unknow	β-TrCP	Blocking PD-L1 from binding to PD- 1 binding	(36)
	Cytarabine	Unknow	GSK3β	Down-regulation of the expression of oncogenic gene SPP1	(58, 59)
	Palbociclib	Palbociclib + PD-1 mAb	CDK4/6	Promotes T cell-mediated anti-tumor immune response	(60)
	Curcumin	Curcumin + CTLA-4 mAb	CSN5	Promotes ubiquitination and degradation of PD-L1	(52)
	PROTACs PD- LYSO	Unknow		PD- L1 degradation	(81–83, 130)
**Phosphorylation**	Metformin	Metformin + CTLA-4 mAb	AMPK	Induced PD-L1 degradation via ERAD pathway	(21, 37, 108)
	SA-49	Unknow	PKCα-GSK3β	Promotes PD-L1 lysosomal degradation	(104)
	Erlotinib, Osimertinib and ES-072	Erlotinib, Osimertinib and ES-072 + PD-1 mAb	EGFR	Induces phosphorylation of non-glycosylated PD-L1	(24, 76, 102, 103)
	Tofacitinib, Ruxolitinib and Fedratinib	Unkonw	JAK1	Downregulation of PD- L1 via suppression of STAT1/3- mediated transcription	(76, 109, 110)
	IL-6 mAb	IL-6 mAb + TIM-3	IL-6	Inhibits downstream JAK1 activation and promotes PD-L1 degradation	(26, 76)
**Acetylation**	Trichostatin A	Trichostatin A+ PD-1 mAb	HDAC2	Inhibition of PD-L1 translocation to the nucleus	(119, 120)
**S-Palmitoylation**	2-BP	Unknow	DHHC family	Decreased palmitoylation and thus increased degradation of PD- L1	(126, 127)

## 5 Discussion

**Figure 2:**
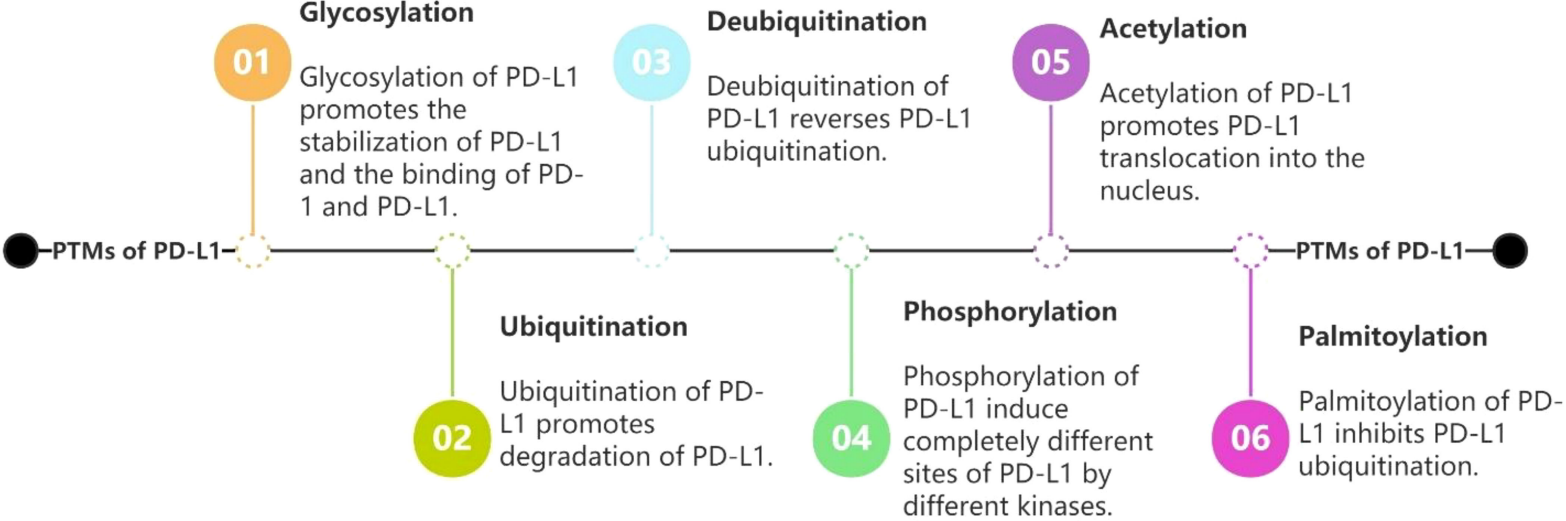
Regulations of PD-L1 by glycosylation, ubiquitination, deubiquitination, phosphorylation, acetylation and palmitoylation.

**Figure 3:**
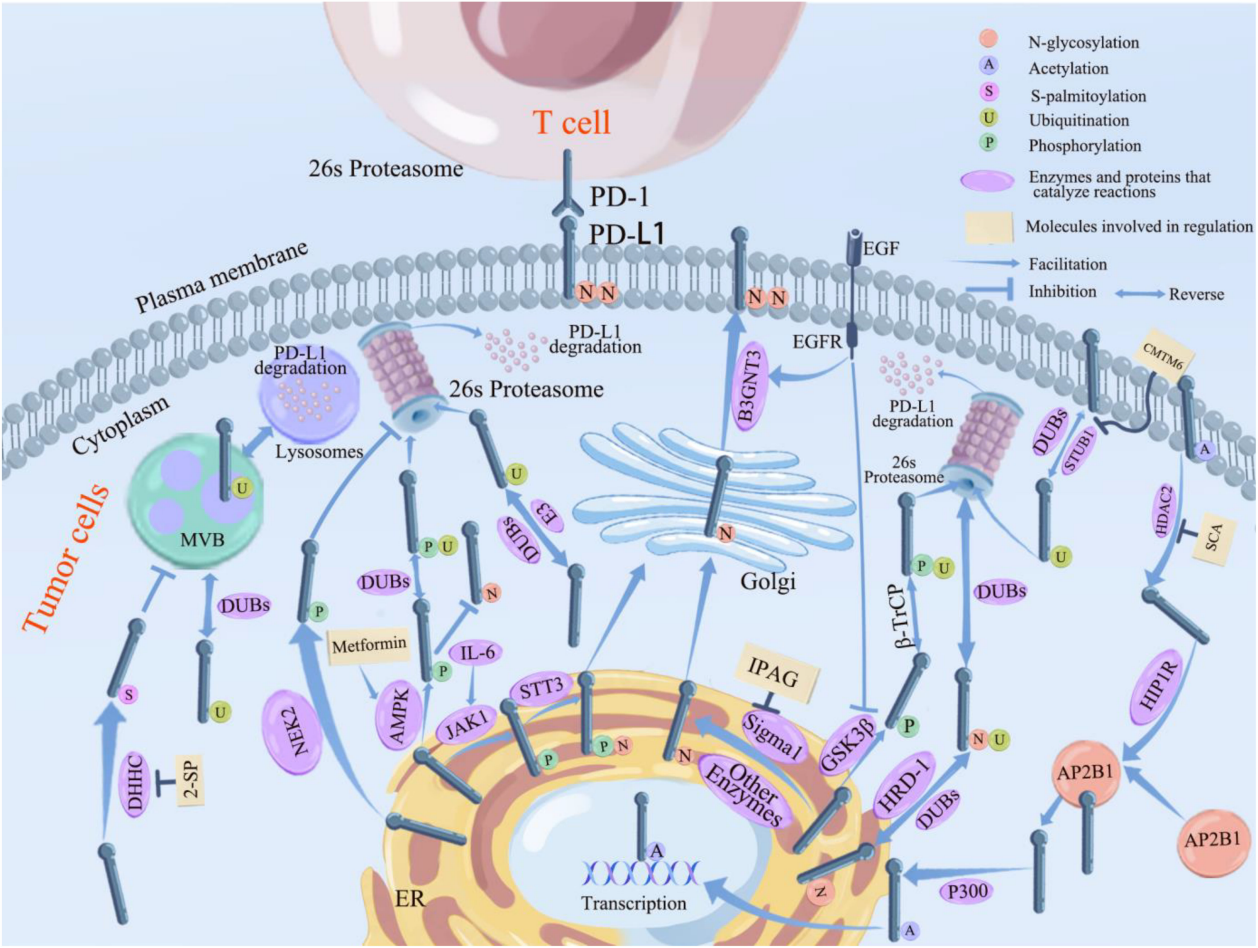
Pathways related to PTMs of PD-L1 in tumor cells and the main related molecules. PD-L1 glycosylation begins in the ER and ends in the Golgi, where it reaches the cell surface and binds to PD-L1 on the surface of T cells, inhibiting lymphocyte function and causing immune escape of tumor cells. STT3, Sigma 1 (which can be inhibited by IPGA), and other enzymes that promote glycosylation (see previous section for details) all promote PD-L1 glycosylation in the endoplasmic reticulum. B3GNT3 (which can be inhibited by EGFR) further promotes the completion of PD-L1 glycosylation in Golgi. Ubiquitination of PD-L1 takes place in the lysosome and 26s proteasome. β-TrCP promotes ubiquitination of PD-L1 after phosphorylation by GSK3β; along with STBU1 (which can be boosted by CMTM6) and other E3 enzymes (see previous section for details on particular enzymes), HRD1 encourages the ubiquitination of abnormally glycosylated PD-L1 so that it can be degraded via the 26s proteasome pathway. Ubiquitination of PD-L1 can be reversed by DUBs including CSN5 and USPs, etc, which inhibitthe degradation of PD-L1. PD-L1 phosphorylated by JAK1 undergoes glycosylation mediates by STT3; PD-L1 phosphorylated by NEK2 inhibits its ubiquitination; PD-L1 phosphorylation mediated by AMPK (which can be inhibited by metformin) inhibits its glycosylation and promotes the degradation of aberrantly glycosylated PD-L1 by ubiquitination; and phosphorylation of PD-L1 mediated by GSK3β (which can be inhibited by EGFR) all promotes it ubiquitination and can be degraded by the 26s proteasome. HDAC2 deacetylates PD-L1 (inhibited by SCA) into the cytoplasm and forms a complex with AP2B1 mediated by HIP1R, which is later reacetylated by P300 and translocated into the nucleus through the action of P300. DHHC promotes PD-L1 palmitoylation and inhibitis its transport to MVB, preventing PD-L1 degradation via the lysosomal pathway.

PD-L1/PD-1 has garnered significant interest in recent years as a signaling pathway that inhibits immune cell activation and promotes immune escape of tumor cells. Given that PTMs are often therapeutic targets for drug-mediated inhibition of cancer, it is crucial to better understand PD-L1 PTMs in malignant tumors. This review summarizes the types of post-translational modifications of PD-L1 that have been identified, the mechanisms underlying regulation of PD-L1 by different post-translational modifications, and the PTMs that have been identified and used to target PD-L1, providing a solid theoretical basis for the improvement of immunotherapeutic effects and combination of drugs. We aim to provide new ideas and directions for tumor immunotherapy research. (See **Figure 2** for the six PTMs of PD-L1. See **Figure 3** for the pathways of PTMs related to PD-L1 in tumor cells and the main related molecules).

PTMs of PD-L1 have made some achievements. Firstly, an increasing number of mechanisms have been translated into clinical drugs (see **Table 2** for details).

Secondly, PD-L1/PD-1 related immunotherapy has more side effects and only some cancer patients respond well to PD-1/PD-L1 blockade, thus a tumor vaccine with PD-L1/PD-1 immunotherapy strategy is proposed to overcome these limitations. Recent studies identified Local tumor photothermal treatment with the near-infrared light at the second window (NIR-II) (131) is a boosting strategy in triggering the *in-situ* tumor vaccination (ISTV) for cancer therapy. It is responsible to reverse the immunosuppressive microenvironment of tumors, increasing the antigen presentation efficacy and promoting the immunological responses of T-cells to attack the remaining tumor cells (131). However, most of the previously developed ISTV adjuvants may indiscriminately damage tumor cells and immune cells, limiting the overall effect of the immune response. Fan et al, designed a “cocktail” nano adjuvant that significantly enhanced the immune response to NIR-II light-induced DC mutation and T-cell differentiation, and had a stronger inhibitory effect on tumor growth (132). Moreover, Hu et al, explored a synergistic strategy to combine *in situ* vaccination and gene-mediated anti-PD therapy. It was generated by unmethylated cytosine-phosphate-guanine (CpG) and pshPD-L1 gene co-delivery. PEI worked as the delivery carrier to co-deliver the CpG and pshPD-L1 genes, the formed PDC (PEI/DNA/CpG) nanoparticles were further shielded by aldehyde modified polyethylene glycol (OHC-PEG-CHO) via pH responsive Schiff base reaction for OHC-PEG-CHO-PEI/DNA/CpG nanoparticles (P(PDC) NPs) preparation (133). In mouse experiments, the synergistic effect of this step was rapid and effective (133).

However, some challenges still remain to be addressed. Firstly, the effects of various PD-L1 PTMs on tumor immune escape mentioned in this paper have been largely clarified, but due to the diversity and complexity of post-translational modification forms and mechanisms, the regulatory mechanisms of PD-L1 PTMs still need to be explored further. For example, the effects of various PTMs on the subcellular localization and physiological functions of PD-L1, and the existence of novel PD-L1 PTMs processes need to be further investigated.

Secondly, most of the corresponding intervention strategies or combination drug regimens for PTM are still in the experimental stage in cellular and animal models. Therefore, future studies will continue to investigate in depth at the mechanistic level on the one hand, and focus on the clinical translation and combination of existing intervention strategies for PD-L1-based PTM on the other hand.

Thirdly, different forms of PTMs mentioned in the text also have their particular breakthrough points.

Glycosylation and Phosphorylation: Glycosylation of PD-L1 inhibits 26S proteasome-mediated protein degradation, which, in turn, maintains PD-L1 stability, and GSK3β is the central node that regulates PD-L1 stability (24). Furthermore, glycosylated PD-L1 inhibits GSK3β phosphorylation and β-TrCP ubiquitination-mediated degradation (99), but the mechanism of the translocation of ER-bound PD-L1 into the cytoplasm and degradation by the 26S proteasome is unclear. Future work may focus on how phosphorylation and glycosylation of PD-L1 regulate the ERAD pathway. In addition, EGFR-activated AKT is associated with cytomembrane PD-L1 expression and survival in patients with lung cancer (134–137). EGFR-activated AKT inhibits GSK3β activity via Ser9 phosphorylation, suppresses EGF signaling in basal-like breast cancer (BLBC) cells, reduces PD-L1 stability, and decreases cancer cell immune escape, thereby demonstrating a therapeutic benefit. Similar regulation was observed by Akbay et al. (138), in PD-L1 mouse lung tumor cells. However, no study has yet indicated whether AKT can directly regulate PD-L1 expression. More in-depth analysis is needed to demonstrate the role of AKT in EGFR-mediated PD-L1 protein stabilization. The effect of the catalysis of ATP uptake mediated by the endoplasmic reticulum on PD-L1 phosphorylation in the endoplasmic reticulum is not understood. Furthermore, the mechanism of how AMPK is localized in the lumen of the endoplasmic reticulum is also unclear. Ubiquitination: Novel small molecule immunotherapeutic agents, PROTACs, generated against PD-L1 ubiquitination have been generated. However, whether these PROTACs can show better clinical outcomes than primary antibodies need to be explored in depth (83). Acetylation: Nuclear PD-L1 expression is higher in metastatic tumors than in primary tumors (118), but the mechanism by which nuclear PD-L1 is increasing the aggressiveness of tumors is currently unclear. Furthermore, whether PD-L1 acetylation is associated with drug resistance is unclear and needs to be explored.

Fourthly, PD-1 modification is also critical in the anti-cancer immune response. However, the mechanisms underlying the regulation of PD-1 PTMs remain largely unknown.

Fifthly, Although PD-L1 has emerged as an important target for drug development, and several approved drugs and related clinical trials targeting PD-L1 are available demonstrating the potential of PD-L1 as a drug target, the response rate is still below 40% in most cancer types. Post-translational modulation of PD-1/PD-L1 and the proposed combination therapeutic strategies to improve the PD-1/PD-L1 blockade efficacy by providing new avenues.

Finally, Most FDA-approved therapeutic antibodies are typically produced by E. coli or other host microorganisms that do not exhibit PTMs. This renders the detection of PD-L1 suboptimal, and therefore, new technologies are needed to improve the efficacy of antibody therapy.

## Author contributions

CF write the article. LZ revised the article. XC and DQ retrieved literature. TZ revised and reviewed the article. All authors contributed to the article and approved the submitted version.
